# Overview of genetic markers for hereditary colorectal cancer

**DOI:** 10.1186/1897-4287-10-S4-A22

**Published:** 2012-12-10

**Authors:** Rodney J Scott

**Affiliations:** 1 The Hunter Medical Research Institute; School of Biomedical Sciences and Pharmacy, University of Newcastle; and the Hunter Area Pathology Service, John Hunter Hospital, Newcastle, New South Wales, Australia

## 

A number of hereditary conditions have been identified that predispose to colorectal cancer. Most inherited forms of colorectal cancer can be placed into two groups, those that are associated with a pre-malignant phenotype (the “polyposis” syndromes) and those do not have a pre-malignant phenotype (generally termed “non-polyposis”). The polyposis syndromes can be further subdivided into two groups; the hamartomatous polyposis syndromes that include Peutz-Jeghers syndrome (PJS), Cowdens’ syndrome (PTEN), juvenile polyposis (JPS) and Ruvalcaba-Myhre-Smith syndrome (RMSS): and the adenomatous polyposis syndromes that include familial adenomatous polyposis, Gardners’ syndrome, Oldfields’ syndrome Turcot syndrome, Carroli’s Disease and MUTYH associated polyposis. The non-polyposis group comprises Lynch syndrome, Muir-Torre’s syndrome and Turcot syndrome (there are two forms of Turcot syndrome, one relating to familial adenomatous polyposis and the other to Lynch syndrome).

The genetic basis of the syndromes listed has revealed disease heterogeneity in the non-polyposis group, where all these syndromes are associated with errors in DNA mismatch repair genes (*MSH2*, *MLH1*, *MSH6* and *PMS2*). With respect to the adenomatous polyposis syndromes most are associated with mutations in the APC gene, the exception being *MUTYH* associated polyposis which has been linked to mutations in *MUTYH.* There are several genes that have been linked to the hamartomatous syndromes, *STK11* (*LKB1*) has been shown to be associated with PJS; *PTEN*, *SMAD4* and *BMPR1A* with JPS and RMSS and JPS.

Variance in disease expression can not be accounted solely by mutations in the respective gene. Differences between and within families harbouring the same mutation suggest that other factors are likely to influence disease risk. In the case of Lynch syndrome it is now becoming clear that a series of modifier genes are linked to the age of disease onset and further, the number of modifier risk alleles appears to increase disease severity. Recent evidence suggests that two modifiers of particular interest, located on chromosomes 8 and 11 significantly influence disease onset in MLH1 mutation carriers.

Knowledge about modifier genes and how they impact on disease expression is important for improving personalized patient care, especially in the setting of life-long surveillance and the potential use of significant prophylactic measures to reduce disease risk.

